# Treating patients with hypertension in Indonesia’s primary health care centre: A challenging condition

**DOI:** 10.21542/gcsp.2019.14

**Published:** 2019-09-20

**Authors:** Lucky Aziza Abdullah Bawazir, Wicensius Sianipar

**Affiliations:** 1 Department of Internal Medicine, Faculty of Medicine, Universitas Indonesia–Cipto Mangunkusumo National Teaching Hospital; 2 Tegal Alur II Community Health Center

## Abstract

Background. In Indonesia, hypertension treatment relies on primary health care services, and there are no current data on blood pressure control and hypertension treatment in Indonesia’s primary health care system.

Methods. We conducted a cross-sectional study including all patients with hypertension (n = 273) who visited the Tegal Alur II Community Health Center in 2017. For all patients with hypertension, medical records containing the most recent blood pressure results and antihypertensive treatments were examined by the authors. The primary outcome in this study was optimal blood pressure control according to the Joint National Committee (JNC) VII guidelines.

Results. The mean systolic and diastolic blood pressures were 143.7 mmHg (SD 18.5) and 87 mmHg (SD 11.4), respectively. Calcium channel blockers were the most frequently used antihypertensive agents (58.6%). Of all patients with hypertension, 27.1% achieved controlled blood pressure. Bivariate analysis revealed a significant association between antihypertensive agents and blood pressure control (p = 0.009). Multivariate analysis showed that calcium channel blockers were the strongest predictor of blood pressure control, with an adjusted odds ratio of 1.9 (95% confidence interval CI [1.1–3.5], *p* = 0.022).

Conclusion. Controlled blood pressure was achieved by less than half of all patients with hypertension visiting the Tegal Alur II Community Health Center in 2017. The majority of patients with hypertension received single-drug therapy with calcium channel blockers, the most frequently used antihypertensive agents. There was a significant association between antihypertensive agents and blood pressure control.

Funding. This study received no financial support from any specific funding agencies.

## Introduction

The high prevalence of hypertension is still a global health burden. In 2013, according to Indonesia’s Ministry of Health, the prevalence of hypertension in Indonesia was 26.5%^1^. Hypertension treatment in Indonesia relies on primary health care services. However, achieving controlled blood pressure is still challenging in Indonesia. According to the Joint National Committee (JNC) in 2003, controlled blood pressure refers to a blood pressure of <140/90 mmHg in people without diabetes and <130/80 mmHg in people with diabetes^2^. A previous study by Hussain showed that only 9% of adults with hypertension achieved controlled blood pressure in Indonesia^3^. A higher rate of controlled blood pressure was found in developed countries such as Denmark (29.1%)^4^. A previous hospital-based study showed that twice-daily administration of antihypertensive drugs was associated with blood pressure control^5^. To our knowledge, little is known about the association between antihypertensive agents and blood pressure control in primary health care. Therefore, it is hypothesized that antihypertensive agents have a significant association with blood pressure control in Indonesia’s primary health care system.

## Methods

### Study design and setting

This cross-sectional study aimed to reveal the association between antihypertensive agents and blood pressure control among patients with hypertension at the Tegal Alur II Community Health Center. This primary health care centre is located in Tegal Alur Administrative Village, Kalideres Subdistrict, West Jakarta. This study was conducted from February 2018 to March 2018.

### Sampling methods

The authors employed a total sampling method. Medical records of all adult patients (aged ≥18 years) with hypertension visiting the Tegal Alur II Community Health Center from January 2017 to December 2017 were included in this study.

### Procedures and instruments

**Figure 1.**
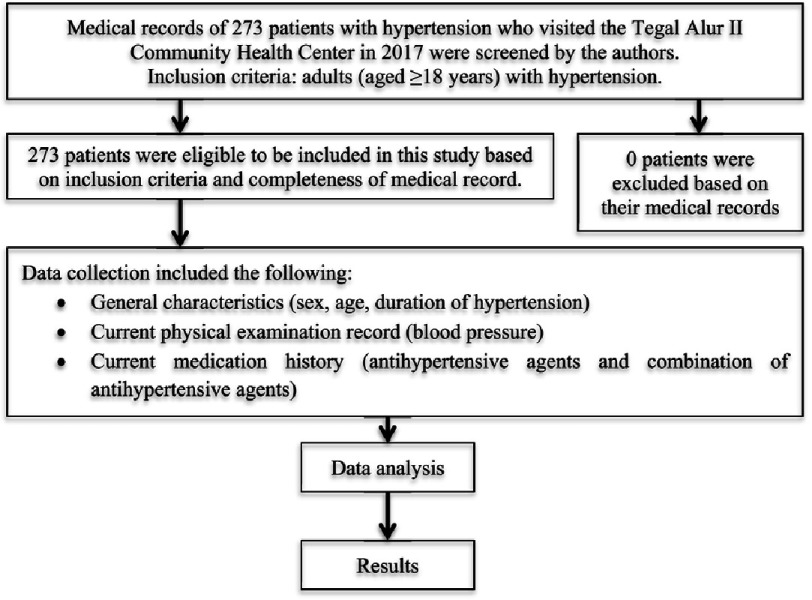
Workflow of the study.

The authors obtained data from the medical records of all patients with hypertension who visited the Tegal Alur II Community Health Center during 2017. Figure 1 provides a summary of the workflow of this study.

### Covariates

The following characteristics of patients with hypertension were recorded: sex, age, duration of hypertension, current blood pressure, comorbidities, antihypertensive agents, and combination of hypertensive agents. Age was categorized as <45 years, 45–65 years, and >65 years. The duration of hypertension was categorized as <1 year, 1–5 years, and >5 years. Comorbidities were divided into two categories: hypertension with diabetes mellitus and hypertension without diabetes mellitus. Antihypertensive agents that were used by physicians in the Tegal Alur II Community Health Center were categorized into the following drug classes: diuretics, angiotensin-converting enzyme (ACE) inhibitors, angiotensin receptor blockers (ARBs), calcium channel blockers (CCBs), and beta blockers. The combination of antihypertensive agents factor was categorized as one drug and two drugs.

### Outcomes

Patients’ blood pressure at Tegal Alur II Community Health Center was measured with a periodically calibrated sphygmomanometer. The outcome measure in this study was blood pressure control, categorized as uncontrolled blood pressure and controlled blood pressure based on the criteria from JNC in 2003. These criteria define controlled blood pressure as <140/90 mmHg in individuals without diabetes and <130/80 mmHg in individuals with diabetes. Blood pressure control data was obtained from mean blood pressure of three latest visits. Primary health care providers in Indonesia adhere to these criteria.

### Data analysis

The categorical data, such as sex, age, duration of hypertension, blood pressure control, comorbidities, antihypertensive agents, and combination of antihypertensive agents, are presented as frequencies (n) and percentages (%). The numerical data, such as age, duration of hypertension, systolic blood pressure (SBP), and diastolic blood pressure, are presented as means and standard deviations (SDs). The multivariate analysis was performed to detect the differences in characteristics between men and women (linear regression for associations of continuous variables with sex; logistic regression for associations of categorical variables with sex). A p-value of <0.05 indicated statistical significance. The distribution of patient characteristics according to comorbidity status (hypertension with and without diabetes mellitus) was also analysed. The bivariate analysis was performed to determine the relationship between treatment (antihypertensive agents and combination of antihypertensive agents) and blood pressure control. The multivariate analysis with a logistic regression model was performed to obtain age- and sex-adjusted odds ratios (ORs) and 95% confidence intervals (95% CIs) for the influence of antihypertensive agents on blood pressure control. The statistical analysis was performed using SPSS version 20.0 (IBM Corp., Armonk, NY).

**Table 1:** Characteristics of patients with hypertension at the Tegal Alur II community health center.

	All subjects N (%) Mean (SD)	Men N (%) Mean (SD)	Women N (%) Mean (SD)	*p*-value^*^
Number of subjects	273 (100.0%)	67 (24.5%)	206 (75.5%)	
Age, mean (SD)	54.7 (SD 10.7)	58.8 (SD 10.8)	53.4 (SD 10.3)	**0.001**
<45 years	48 (17.6%)	7 (10.4%)	41 (19.9%)	–
45–65 years	185 (67.8%)	46 (68.7%)	139 (67.5%)	–
>65 years	40 (14.7%)	14 (20.9%)	26 (12.6%)	–
Duration of hypertension (in years), mean (SD)	0.8 (SD 1.7)	0.5 (SD 0.7)	0.9 (SD 1.8)	0.064
<1 year	161 (59%)	41 (61.2%)	120 (58.3%)	–
1–5 years	107 (39.2%)	26 (38.8%)	81 (39.3%)	–
>5 years	5 (1.8%)	0 (0.0%)	5 (2.4%)	–
Blood pressure (in mmHg)				
SBP, mean (SD)	143.7 (SD 18.5)	143.7 (SD 16.9)	143.7 (SD 19.0)	0.936
DBP, mean (SD)	87.0 (SD 11.4)	86.7 (SD 10.2)	87.6 (SD 10.1)	0.891
Blood pressure control				0.607
Uncontrolled	199 (72.9%)	50 (74.6%)	149 (72.3%)	–
Controlled	74 (27.1%)	17 (25.4%)	57 (27.7%)	–
Comorbidities				0.751
Hypertension with diabetes mellitus	12 (4.4%)	3 (4.5%)	9 (4.4%)	–
Hypertension without diabetes mellitus	261 (95.6%)	64 (95.5%)	197 (95.6%)	–
Antihypertensive agents				
Diuretics	1 (0.4%)	0 (0.0%)	1 (0.5%)	1.0
ACE inhibitors	128 (46.9%)	29 (43.4%)	99 (48.1%)	0.956
ARBs	5 (1.8%)	1 (1.5%)	4 (1.9%)	0.956
CCBs	160 (58.6%)	43 (64.2%)	117 (56.8%)	0.967
Beta blockers	4 (1.5%)	1 (1.5%)	3 (1.5%)	1.0
Combination of antihypertensive agents				0.949
1 drug	248 (90.8%)	60 (89.6%)	188 (91.3%)	–
2 drugs	25 (9.2%)	7 (10.4%)	18 (8.7%)	–

**Notes.**

N Number SD Standard deviation SBP Systolic blood pressure DBP Diastolic blood pressure ACE Angiotensin-converting enzyme ARB Angiotensin receptor blocker CCB Calcium channel blocker

* Linear regression was performed to detect associations of continuous (numerical) variables with sex, while logistic regression was performed to detect associations of categorical variables with sex.

**Table 2:** Distribution of patients’ characteristics according to comorbidities of hypertension.

	Hypertension with diabetes mellitus N (%) Mean (SD)	Hypertension without diabetes mellitus N (%) Mean (SD)
Number of subjects	12 (4.4%)	261 (95.6%)
Age (in years), mean (SD)	59.6 (SD 11.5)	54.5 (SD 10.6)
<45 years	0 (0.0%)	48 (18.4%)
45–65 years	10 (83.3%)	175 (67%)
>65 years	2 (16.7%)	38 (14.6%)
Duration of hypertension (in years, mean (SD)	0.58 (SD 0.51)	0.82 (SD 1.7)
<1 year	5 (41.7%)	156 (59.8%)
1–5 years	7 (58.3%)	100 (38.3%)
>5 years	0 (0.0%)	5 (1.9%)
Blood pressure (in mmHg)		
SBP, mean (SD)	134.2 (SD 12.5)	144.1 (SD 18.2)
DBP, mean (SD)	81.7 (SD 12.7)	87.3 (SD 11.3)
Blood pressure control		
Uncontrolled	6 (50.0%)	193 (73.9%)
Controlled	6 (50.0%)	68 (26.1%)
Antihypertensive agents		
Diuretics	0 (0.0%)	1 (0.4%)
ACE inhibitors	3 (25%)	125 (47.9%)
ARBs	0 (0.0%)	5 (1.9%)
CCBs	9 (75.0%)	151 (57.9%)
Beta blockers	0 (0.0%)	4 (1.5%)
Combination of antihypertensive agents		
1 drug	12 (100.0%)	236 (90.4%)
2 drugs	0 (0.0%)	25 (9.6%)

**Notes.**

N Number SD Standard deviation SBP Systolic blood pressure DBP Diastolic blood pressure ACE Angiotensin-converting enzyme ARB Angiotensin receptor blocker CCB Calcium channel blocker

## Results

### Characteristics of patients with hypertension

In total, 273 patients with hypertension were included in this study. Table 1 provides the distribution of patient characteristics according to sex. More than half of the patients were women (75.5%). The mean age of the subjects in this study was 54.7 years (SD 10.7 years). From the multivariate analysis, the only significant difference between men and women was age. The mean age of men was significantly higher (*p* = 0.001) than that of women (58.8 years, SD 10.8, and 53.4 years, SD 10.3, respectively). The largest age group in this study was 45–65 years (67.8%). Most patients had hypertension for less than 1 year (59.0%). The mean SBP in this study was higher than the blood pressure target recommendation (143.7 mmHg, SD 18.5). Controlled blood pressure (<140/90 mmHg for patients without diabetes and <130/80 for patients with diabetes) was achieved by 27.1% of the patients. Most of the patients did not have diabetes mellitus (95.6%). CCBs were the most frequent antihypertensive agents used by the subjects in this study (58.6%). Most of the patients received single-drug therapy (90.8%) as their hypertension treatment.

Table 2 provides a summary of the patient characteristics according to hypertension comorbidities. The mean age of patients with hypertension and comorbid diabetes mellitus was higher than that of patients with hypertension without diabetes mellitus (59.6 years, SD 11.5, and 54.5 years, SD 10.6, respectively). The majority of patients with diabetes mellitus had hypertension for 1–5 years (58.3%), and the majority of patients without diabetes mellitus had hypertension for <1 year (59.8%). For both groups, the mean SBP was above the target recommendation; the mean SBP was 134.2 mmHg (SD 12.5) in the hypertension with diabetes mellitus group and 144.1 mmHg (SD 18.2) in the hypertension without diabetes mellitus group. Most patients in the diabetes and non-diabetes groups had uncontrolled blood pressure (50.0% and 73.9%, respectively). More than half of the patients in the diabetes and non-diabetes groups received CCBs (75.0% and 57.9%, respectively). All patients in the diabetes group received single-drug treatment. Additionally, most patients in the non-diabetes group received single-drug treatment (90.4%).

### Antihypertensive agents and blood pressure control

Table 3 provides the distribution of antihypertensive agents according to blood pressure control. In patients with uncontrolled blood pressure and those with controlled blood pressure, CCBs (55.8% and 66.2%, respectively) were the most frequently used antihypertensive agents, followed by ACE inhibitors (52.3% and 32.4%), ARBs (1.5% and 2.7%), beta blockers (0.5% and 4.1%), and diuretics (0.5% and 0.0%). There was a significant association between antihypertensive agents and blood pressure control (*p* = 0.009). The majority of patients in the uncontrolled blood pressure group and in the controlled group received single-drug treatment (89.4% and 94.6%, respectively). There was an insignificant association between combination of antihypertensive agents and blood pressure control (*p* = 0.190).

**Table 3:** Distribution of antihypertensive agents and blood pressure control.

	Uncontrolled blood pressure N (%)	Controlled blood pressure N (%)	*p*-value
Antihypertensive agents			**0.009**
Diuretics	1 (0.5%)	0 (0.0%)	
ACE inhibitors	104 (52.3%)	24 (32.4%)	
ARBs	3 (1.5%)	2 (2.7%)	
CCBs	111 (55.8%)	49 (66.2%)	
Beta blockers	1 (0.5%)	3 (4.1%)	
Combination of antihypertensive agents			0.190
1 drug	178 (89.4%)	70 (94.6%)	
2 drugs	21 (10.6%)	4 (5.4%)	

**Notes.**

ACE Angiotensin-converting enzyme ARB Angiotensin receptor blocker CCB Calcium channel blocker

Table 4 provides age- and sex-adjusted ORs of the influence of associated variables on controlled blood pressure. The strongest predictor of controlled blood pressure among the patients with hypertension in the Tegal Alur II Community Health Center was treatment with CCBs (OR: 1.9, 95% CI [1.1–3.5], *p* = 0.022).

**Table 4:** Associations of independent variables with blood pressure control based on a logistic regression model.

	Controlled blood pressure	
	OR (95% CI)	*p*-value
Diabetes mellitus	2.4 (0.7–7.9)	0.145
Antihypertensive agents		
Diuretics	4.1 (0.0–0.0)	1.0
ACE inhibitors	4.4 (0.5–34.9)	0.160
ARBs	0.0 (0.0–0.0)	0.999
CCBs	**1.9 (1.1–3.5)**	**0.022**
Beta blockers	1.3E+10 (0.0–0.0)	0.999
Combination of antihypertensive agents	4.4 (0.5–34.9)	0.160

**Notes.**

The reference group was “uncontrolled blood pressure”.

ACE Angiotensin-converting enzyme ARB Angiotensin receptor blocker CCB Calcium channel blocker

## Discussion

In this study, 27.1% of patients with hypertension achieved controlled blood pressure. This finding was higher than that from a previous study by Hussain et al.^3^ in Indonesia, which stated that only 9% of patients with hypertension achieved controlled blood pressure. This result was also similar to the result from a study of Denmark’s primary health care system, in which 29.1% of all patients achieved controlled blood pressure^4^. This study showed that the rate of blood pressure control was the highest in patients with hypertension aged >65 years (35%). With the consideration that this study population was dominated by women (75.5%), a study by Llisterri et al.^6^ showed that 3 out of 10 women aged >65 years achieved controlled blood pressure, which was similar to this study’s finding for patients with hypertension aged >65 years. In this study, half of patients with hypertension and comorbid diabetes mellitus achieved controlled blood pressure. This frequency was higher than the rate of blood pressure control among hypertensive patients with diabetes in the Danish primary health care system^7^.

The mean SBP of patients with hypertension and comorbid diabetes (134.2 mmHg, SD 12.5) and without diabetes (144.1 mmHg, SD 18.2) in this study was above the target blood pressure recommended by JNC VII. The mean SBP among patients with hypertension and comorbid diabetes in this study was higher than the mean SBP of 131 mmHg among patients with diabetes in a study of the Irish primary health care system^8^.

The mean SBP among patients with hypertension without diabetes mellitus in this study was also higher than the mean SBP of 136 mmHg among patients with hypertension without diabetes mellitus in the study of the Irish primary health care system^8^. The mean SBP in this study (143.7 mmHg, SD 18.5) was better than the result from a study by Didier and Guimaraes^9^ in Brazil (158.6 mmHg, SD 9.9). This evidence shows that patients in developed countries still more frequently achieve target blood pressure than patients in developing countries. However, few diabetes patients visit primary health care centres in Indonesia, as the majority of diabetes patients visit secondary and tertiary health care centres due to adverse clinical manifestations resulting from uncontrolled blood glucose and blood pressure.

The most frequently used class of antihypertensive agents in this study was CCBs (58.6%). This result was similar to the result from a study by Mori et al.^10^ in Japan, which showed that 50.3% of all patients with hypertension received CCBs. This result was also supported by Chia et al.^11^, who showed that the most frequently used class of antihypertensive agents in a Malaysian primary care setting was CCBs (62.6%). This result differed from that of a study in a primary care setting by Novello et al.^12^, which showed that the most frequently used class of antihypertensive agents was diuretics. Another study by Abegaz et al.^5^ also showed that diuretics (thiazide) were the most frequently used antihypertensive drugs in patients with hypertension. This study’s result was also different from the findings of a meta-analysis in Brazil, in which ACE inhibitors were the most frequently used antihypertensive agents in primary care practices^13^. Every primary care setting has its own preferred first-line treatment while still adhering to the recommendation by JNC VII^2^.

This study showed that in patients with hypertension and comorbid diabetes, CCBs were the most frequently used antihypertensive agents (75%). The results of this study differed from those of the Malaysian primary care study by Cheong et al.^14^, in which ACE inhibitors were the antihypertensive agents most frequently prescribed to patients with hypertension and diabetes mellitus. This result is supported by the JNC VII guidelines, which state that the first-line treatment options for patients with hypertension and diabetes mellitus are thiazide, ACE inhibitors, beta blockers, CCBs, or ARBs^2^. However, the most beneficial antihypertensive agents for reducing the progression of diabetic nephropathy in diabetes patients are ACE inhibitors and ARBs^2,15^.

In this study, all patients with hypertension and diabetes mellitus received single-drug therapy. In contrast to this study’s finding, a recommendation by the American Diabetes Association stated that multidrug therapy should be used to achieve controlled blood pressure in people with diabetes^16^. A study by Cheong et al.^14^ also showed that the number of drug combinations had a significant positive association with blood pressure control. This evidence could explain the failure of the hypertension with diabetes mellitus group in this study to achieve a mean SBP below 130 mmHg, as 50% of the patients in this group had uncontrolled blood pressure.

In this study, antihypertensive agents had a significant association with blood pressure control. CCBs were more frequently used by patients with controlled blood pressure than by patients with uncontrolled blood pressure (66.2% and 55.8%, respectively). This result was in contrast to the result by Alba-Leonel et al.^17^, which showed that CCBs had a lower rate of use among individuals with controlled blood pressure than among those with uncontrolled blood pressure (4.8% vs 6.8%, respectively), although that result was insignificant. After adjusting for age, sex, and comorbidities (diabetes mellitus), CCBs were the strongest predictor of blood pressure control at the Tegal Alur II Community Health Center. In contrast to this study, a study by Bronsert et al.^18^ showed that ACE inhibitors and beta blockers had significantly higher rates of blood pressure control than CCBs. Contrary to the findings in the study by Bronsert et al.^18^, which showed that multidrug therapy had better blood pressure control than single-drug therapy, the bivariate and multivariate analyses in this study showed no significant association between combinations of antihypertensive agents and blood pressure control.

The limitation of this study was that there was no blood pressure change measurement to evaluate the effectiveness of antihypertensive agents in reducing blood pressure. Additionally, this study evaluated office blood pressure and did not produce conclusive results regarding the association of treatment type with blood pressure changes. Further study is needed to evaluate other determinants related to antihypertensive agents and blood pressure control, such as the treatment adherence and compliance of patients with hypertension. Nevertheless, this is the first study to evaluate the relationship between antihypertensive agents and blood pressure control in an Indonesian primary health care practice. General physicians in Indonesian primary health care settings are recommended to use CCBs and other first-line antihypertensive agents when treating patients with hypertension. Due to the difficulties in achieving controlled blood pressure in people with diabetes, ACE inhibitors or ARBs and a combination of antihypertensive agents are recommended treatments for patients with hypertension and comorbid diabetes.

## Conclusions

Controlled blood pressure was achieved by less than half of all patients visiting the Tegal Alur II Community Health Center in 2017. The majority of patients with hypertension received single-drug therapy with CCBs, the most frequently used class of antihypertensive agent. CCBs were the strongest predictor of blood pressure control at the Tegal Alur II Community Health Center. There was a significant association between antihypertensive agents and blood pressure control.

## Disclosure

### Ethical approval and consent to participate

This study protocol was approved by the Health Research Ethics Committee, Faculty of Medicine, Universitas Indonesia–Cipto Mangunkusumo Hospital with the reference number of the following: 0549/UN2.F1/ETIK/2018. Because this study used medical records as secondary data and had no direct contact with patients, informed consent was not obtained.

### Availability of data and materials

All raw data of this study are included in this published article. The authors are keen to collaborate in answering further research questions and to participate in systematic reviews or metaanalysis. No additional data are available.

## Competing interests

The authors have no conflict of interest to declare.

### Funding

This study did not receive specific financial support from any funding organization.

## LIST OF ABBREVIATIONS

SD: Standard deviation
OR: Odds ratio
SBP: Systolic blood pressure
DBP: Diastolic blood pressure
ACE: Angiotensin-converting enzyme
ARB: Angiotensin receptor blocker
CCB: Calcium channel blocker
